# Development and psychometric validation of the Amateur Football Tournament Satisfaction Scale using Rasch analysis

**DOI:** 10.3389/fpsyg.2026.1952185

**Published:** 2026-10-14

**Authors:** Xingsheng Zhou, Kai Deng, Ziyuan Wen, Xiancheng Zeng

**Affiliations:** 1 Football College, Guangzhou Sport University, Guangzhou, China; 2 Faculty of Health Sciences and Sports, Macao Polytechnic University, Macau, Macao SAR, China

**Keywords:** amateur football, psychometric validation, Rasch model, scale development, tournament satisfaction

## Abstract

**Introduction:**

Amateur football tournaments attract millions of participants worldwide, yet no dedicated instrument exists to measure players’ satisfaction with these experiences. This study developed and validated the Amateur Football Tournament Satisfaction Scale in full-length and short-form versions using Rasch analysis across two independent samples.

**Methods:**

In Study 1, we generated an initial 30-item pool and used factor and Rasch analyses with 245 amateur players to purify the items, producing a 26-item full-length scale and a 10-item short form. In Study 2, we independently validated the short form with 407 participants through confirmatory factor analysis, reliability and validity testing, and differential item functioning analysis.

**Results:**

Both studies supported a unidimensional structure, with three purification rounds yielding the 26-item scale and confirmatory factor analysis supporting the short form’s structure. The short form demonstrated high internal consistency (*α* = 0.897) and expected correlations with training satisfaction, group cohesion, and burnout. It correlated at 0.974 with the full-length version, although this may partly reflect item overlap. Most items functioned equivalently across gender and league participation groups, but a few Study 1 items showed significant DIF.

**Discussion:**

The Amateur Football Tournament Satisfaction Scale offers a psychometrically sound instrument for assessing amateur football tournament satisfaction. The full-length version suits detailed research, while the short form provides an efficient option for routine evaluation.

## Introduction

Amateur football leagues have gained global popularity over recent decades, with many registered players worldwide participating at the recreational level. Beyond its immediate sporting context, amateur football participation in China is embedded within broader state-led initiatives that link sport to national development, social cohesion, and civic engagement (Jiang and Bairner, 2020; Sullivan et al., 2019). Participant satisfaction with tournament experience acts as a key predictor of sustained participation and long-term athlete loyalty (Armbrecht and Andersson, 2020). However, existing satisfaction measures target elite athletes (Riemer and Chelladurai, 1998) or general leisure contexts (Beard and Ragheb, 1980), neither of which adequately captures the competition-specific dimensions relevant to amateur football. This study addresses this gap by developing and validating the Amateur Football Tournament Satisfaction Scale (AFTSS) using a Rasch model analysis across two independent participant samples.

### Theoretical foundation, experiential framework, and research gaps

Existing satisfaction measurement tools in sport contexts fall short of addressing the unique characteristics of amateur football tournament participation. Three specific gaps warrant attention in the current study. The first gap concerns the absence of a football-specific tournament satisfaction measure. Existing instruments were developed for elite athletes (Riemer and Chelladurai, 1998) or general leisure contexts (Beard and Ragheb, 1980), and even the Tournament Satisfaction Scale (Öcal, 2016), while tournament-oriented, was validated across multiple sports without football-specific calibration. Emerging evidence highlights the importance of football-specific psychosocial factors in shaping children’s participation behavior (Zeng et al., 2026a,b), yet no dedicated satisfaction instrument exists for this population. The second gap concerns methodology, as no instrument in this domain has undergone Rasch-based item-level scrutiny, leaving questions of unidimensionality, item fit, and differential item functioning unanswered. The third gap concerns instrument flexibility, with researchers and practitioners lacking paired full-length and short-form options that accommodate both comprehensive academic investigations and efficient routine evaluations. The present study was designed to address these three gaps sequentially through two independent substudies.

### A unidimensional conceptualization with five content domains

This study draws upon leisure satisfaction theory as its primary theoretical framework (Beard and Ragheb, 1980), leisure satisfaction as a global evaluative judgment that integrates inputs from multiple experiential sources. While satisfaction derives from diverse aspects of the leisure experience, the construct itself operates as a unified, overarching evaluation rather than a collection of independent subdimensions. This integration is particularly evident for the social identity domain, where football participation has been shown to serve as a vehicle for expressing collective identity and cultural belonging (Lee, 2022), suggesting that seemingly distinct experiential domains converge into a unified evaluative judgment. Recent Rasch-based validation of the Leisure Satisfaction Scale short form further confirmed the unidimensional structure of leisure satisfaction, supporting the treatment of satisfaction as a single overarching construct (Kim and Cho, 2022). To ensure comprehensive content coverage, we identified five content domains from the existing literature that collectively represent the range of experiences relevant to amateur football tournament participants. These domains served as item-generation sources rather than hypothesized subdimensions of the scale.

The first of these concerns venue environment and tangible facilities. Grounded in the service quality literature, tangible environmental attributes constitute the most immediately perceptible aspect of any service experience. Empirical research in grassroots sport contexts has confirmed that venue tangible quality functions as a distinct predictor of overall satisfaction (Lianopoulos et al., 2024; Magnusen et al., 2023). Beyond tangible conditions, the tournament experience is fundamentally shaped by affective enjoyment and emotional responses. Participation in amateur football tournaments generates a range of emotional states including excitement, joy, and stress relief, and hedonic enjoyment contributes uniquely to participants’ satisfaction judgments (Scanlan et al., 1993). Social identity and team belonging constitute a third domain. Amateur football is inherently a social activity in which team membership and interpersonal relationships form central elements of the experience (Benson and Bruner, 2018). The fourth domain encompasses tournament organization and service quality, including registration procedures, match scheduling, referee assignments, and safety protocols (Öcal, 2016; Riemer and Chelladurai, 1998). These administrative and logistical elements, though less emotionally salient than the preceding domains, critically influence participants’ overall evaluation. The fifth and final domain addresses personal development and self-realization, drawing on self-determination theory (Deci and Ryan, 2000) and the distinction between hedonic enjoyment and long-term athletic growth (Chen et al., 2019; Rodrigues et al., 2024). This dimension captures the eudaimonic aspect of tournament participation, reflecting players’ perceptions of meaningful progress and competence development.

This conceptualization rests on a central premise of leisure satisfaction theory that individuals form a global evaluative judgment that synthesizes diverse inputs into a unified perception. For amateur football participants, satisfaction with venue conditions, emotional responses, social connections, organizational quality, and developmental outcomes thus coalesces into a single overarching evaluation rather than remaining independent subdimensions. This premise justifies treating tournament satisfaction as a unidimensional construct while still generating items from multiple content areas to ensure comprehensive content validity.

### Rasch measurement theory as an analytical framework

The development of the Amateur Football Tournament Satisfaction Scale presented a three-fold methodological challenge. Generating items across five content domains while verifying a single overarching satisfaction construct represented the first task. Producing both a full-length version and an efficient short form without compromising measurement precision constituted the second. Confirming that the scale functioned equivalently across gender and league participation groups formed the third. Classical test theory, the conventional framework for sport and leisure scale development, cannot adequately address these interrelated demands (Pierobon and Krägeloh, 2026). Its item parameters are sample-dependent, its total scores treat ordinal responses as interval data, and it offers no empirical test of unidimensionality (Hergesell, 2022; Tenenbaum et al., 2007).

The Rasch model resolves each of these limitations, which transforms ordinal Likert responses into interval-level logit measures, placing persons and items on a common linear continuum (Wright and Masters, 1982). This property is essential for developing a short form that captures information comparable to the full-length version. The model also provides item-level fit statistics that identify misfitting items for targeted removal (Bond, 2015; Chen et al., 2026), ensuring that retained items define a coherent measurement continuum. Rasch analysis further tests unidimensionality empirically through principal component analysis of standardized residuals (Dong et al., 2024; Murero and Rice, 2013), directly addressing our premise that satisfaction sampled from multiple domains nevertheless operates as a unified global judgment. Finally, differential item functioning analysis examines whether items operate equivalently across subgroups (Hwang et al., 2025), a prerequisite for valid group comparisons in amateur football populations where gender and competitive experience vary widely.

These properties make the Rasch model the preferred framework for this investigation. It is the only commonly available psychometric approach that simultaneously enables item-level diagnosis, empirical unidimensionality testing, cross-group fairness evaluation, and defensible short-form development. This methodological alignment between the analytical framework and our three substantive objectives provides the foundation for the two-substudy design reported below.

### The present study

The current study aimed to develop and validate both full-length and short-form versions of the Amateur Football Tournament Satisfaction Scale (AFTSS) using Rasch model analysis. The research proceeded through two independent substudies with separate participant samples. Substudy 1 focused on scale development and initial item purification. We generated an initial pool of 30 items covering the five content domains identified above. Using exploratory factor analysis and Rasch rating scale model analysis with a sample of 245 amateur football players, we purified the item pool through three iterative rounds to produce a 26-item full-length AFTSS. Substudy 2 aimed to derive and independently validate a 10-item short form using a new sample of 407 participants. We conducted confirmatory factor analysis to examine the unidimensional factor structure of the short form, evaluated internal consistency and criterion-related validity through correlations with established satisfaction measures, and assessed measurement fairness across gender and league participation groups using Rasch-based differential item functioning.

By addressing the identified gaps in the literature, this study contributes a dedicated, psychometrically rigorous measurement tool for amateur football tournament satisfaction that is suitable for both academic research and practical event evaluation. The availability of paired full-length and short-form instruments provides researchers and practitioners with flexible options tailored to their specific assessment needs.

## Study 1: exploratory factor analysis for AFTSS

Study 1 aimed to develop an initial item pool and examine the factor structure of the Amateur Football Tournament Satisfaction Scale. A total of 30 items were generated based on a comprehensive literature review covering five content domains of the amateur football tournament experience. Exploratory factor analysis (EFA) and Rasch model analysis were then employed to examine the underlying structure of the scale and to purify the item pool (Carpenter, 2018). For this purpose, a sample of amateur football players was recruited from three types of tournaments in China.

Preliminary measurement fairness of the scale was also assessed by examining differential item functioning across gender and age groups. The overarching goal was to establish a psychometrically sound full-length version of the AFTSS for subsequent validation.

### Methods

#### Sample

Participants were recruited from three types of amateur football tournaments in China: provincial-level official leagues, municipal tiered leagues, and tourism-integrated commercial leagues. Stratified random sampling ensured representation across age, playing experience, and team types. A total of 858 amateur football players completed the initial survey. We applied three exclusion criteria: (a) we excluded 387 participants who failed instructed response items embedded in the questionnaire; (b) we retained only participants who reported prior participation in organized amateur league competitions, resulting in the exclusion of 67 participants who had never played in such settings; and (c) we excluded 159 participants who showed invariant response patterns, defined as selecting the same response option across all items in a scale (Huang et al., 2012).

The final sample comprised 206 male participants (84.1%) and 39 female participants (15.9%), with ages ranging from 18 to 45 years (*M* = 22.4, *SD* = 4.1). The sample size of 245 exceeded the minimum recommended sample size of 200 for Rasch analysis with 30 items.

### Measures

#### Amateur Football Tournament Satisfaction Scale (AFTSS) initial item pool

We developed an initial pool of 30 items covering five content domains of the amateur football tournament experience: venue environment, affective enjoyment, social identity and belonging, tournament organization, and personal development. The selection of these five domains was informed by the leisure satisfaction framework and the sport service quality literature, as detailed in the Introduction. We generated items based on a comprehensive literature review of existing satisfaction measures, drawing on established item wording and content coverage from the Athlete Satisfaction Questionnaire (Riemer and Chelladurai, 1998), the Leisure Satisfaction Scale (Beard and Ragheb, 1980), and more recent amateur sport satisfaction research. Following the domain definitions, we developed six items for each domain, resulting in a 30-item initial pool. Each item was rated on a 5-point Likert scale ranging from 1 (strongly disagree) to 5 (strongly agree). We also included an instructed response item asking participants to select “strongly agree.”

To assess content validity, we invited eight experts to evaluate the relevance and clarity of the 30 items (Lambert and Newman, 2023). The expert panel included university faculty specializing in football pedagogy and sport psychology, football club coaches, and administrators from provincial football associations and regional football development centers. All experts had direct experience with amateur football competitions or grassroots player development. Each expert was asked to rate every item on a 5-point scale for both relevance to the target construct and clarity of wording. Item-level content validity indices were then calculated for each item, defined as the proportion of experts who rated the item as 3 or higher on the 5-point scale. All 30 items achieved I-CVI values of 1.00. The scale-level content validity indices were also excellent, with S-CVI/Ave = 1.00 and S-CVI/UA = 1.00, exceeding the recommended thresholds of 0.90 and 0.80, respectively, (Boateng et al., 2018; Polit and Beck, 2006). These results confirmed that the initial item pool had strong content validity.

#### Demographic variables

Demographic information was collected, including age, gender, and years of formal football training. Participants reported their gender (1 = male, 2 = female), age in years, and the total number of years they had engaged in organized football training. Questions also addressed whether participants held a formal football athlete certification and whether they had prior experience participating in organized amateur leagues, with examples provided including corporate leagues, class-based or university-level competitions, tourism-integrated commercial leagues, provincial super leagues, and municipal super leagues (1 = yes, 2 = no). Participants who responded “no” to the league experience question were excluded from the final sample, as the target population of this study was amateur football players with actual tournament participation experience. Additionally, participants reported the type of tournament in which they were currently competing.

### Procedures

Ethical approval for this study was obtained from the institutional review board (IRB: 2026LCLL-097). Online questionnaires were distributed between June and July 2026. Two channels were used to approach participants: sport science students enrolled in university programmes and amateur league players registered in the three tournament types. Potential participants were contacted via university course coordinators and league team captains, who shared the survey link through institutional communication platforms and team social media groups.

The survey was administered through a widely used Chinese online survey platform (*Wenjuanxing*). The questionnaire opened with an informed consent statement that explained the purpose of the study, the voluntary nature of participation, and the confidentiality of all responses. Participants provided electronic consent by clicking “I agree to participate” before accessing the questionnaire items. The survey was designed to take approximately 8 to 10 min to complete. No monetary compensation or course credit was offered for participation.

### Data analysis

All analyses were conducted using SPSS 26.0, Mplus 8.4, and WINSTEPS 5.6.0.0. Descriptive statistics including means, standard deviations, skewness, and kurtosis were first examined for each of the 30 items. We assessed the normality of the data distribution and checked for missing values.

EFA was conducted in Mplus using principal axis factoring with Geomin oblique rotation. Model fit was evaluated with CFI, TLI, RMSEA, and SRMR (Worthington and Whittaker, 2006). The number of factors to retain was determined through parallel analysis, which compares the eigenvalues extracted from the actual data against those derived from randomly generated data. Factors with eigenvalues exceeding the 95th percentile of the random eigenvalues were retained for interpretation. Items were considered satisfactory if they achieved factor loadings of 0.40 or higher on their primary factor and showed no cross-loadings within 0.20 of the primary loading.

The data were fitted to the Rasch rating scale model, which is appropriate for Likert-scale items with ordered response categories. Item fit was evaluated using infit and outfit mean-square statistics, with values between 0.5 and 1.5 considered acceptable. Items with fit statistics outside this range were candidates for removal. Three iterative rounds of item purification were conducted. In each round, the poorest-fitting items were removed, and the model was re-estimated with the remaining items. After each round, the unidimensionality assumption was also examined through principal component analysis of the standardized residuals, with the scale considered unidimensional if the first contrast eigenvalue fell below 2.0 and the raw variance explained by the measures exceeded 50 percent. The item purification process continued until all remaining items met the fit criteria and the unidimensionality conditions were satisfied.

Finally, we conducted preliminary differential item functioning (DIF) analysis using the Mantel–Haenszel method to examine whether items operated differently across gender and age groups. Given the uneven subgroup sizes in this exploratory sample (male *n* = 206, female *n* = 39; younger age group *n* = 189, older age group *n* = 56), we interpreted these DIF results cautiously and treated them as preliminary evidence to be further examined in Study 2 with a larger, more balanced sample.

## Results

### Descriptive statistics and exploratory factor analysis

We first examined the descriptive statistics for all 30 items. Items ranged from 3.74 to 4.37, and standard deviations ranged from 0.65 to 0.93. Skewness values ranged from −1.33 to −0.62, and kurtosis values ranged from 0.37 to 2.90, indicating acceptable univariate normality for all items.

We conducted exploratory factor analysis using principal axis factoring with Geomin oblique rotation. The optimal model was determined through parallel analysis, which compares the eigenvalues from the actual data against those from randomly generated data. As shown in Table 1, the eigenvalue for the first factor from the actual data was 16.96, which exceeded the 95th percentile criterion value of 1.82. The eigenvalues for the second through sixth factors did not exceed their corresponding random criteria. This result suggested that a single factor structure adequately represented the data. The single factor accounted for 42.7% of the total variance.

**Table 1:** Results of different factors model of EFA.

Model	χ^2^	*df*	RMSEA	CFI	TLI	SRMR	Actual eigenvalue	95th percentile criterion value	Decision
One Factor	888.914	405	0.070	0.876	0.867	0.050	16.962	1.824	Retain
Two Factors	793.572	376	0.067	0.893	0.876	0.042	1.335	1.689	Reject
Three Factors	663.775	348	0.061	0.919	0.899	0.033	1.265	1.597	Reject
Four Factors	596.404	321	0.059	0.930	0.904	0.028	1.007	1.527	Reject
Five Factors	506.320	295	0.054	0.946	0.920	0.024	0.792	1.462	Reject
Six Factors	474.064	270	0.056	0.948	0.916	0.022	0.750	1.406	Reject

Table 2 presents the fit indices for alternative factor solutions. The one-factor model produced the following fit statistics: *χ*^2^(405) = 888.91, RMSEA = 0.070, CFI = 0.876, TLI = 0.867, SRMR = 0.050. We then examined the Geomin rotated factor loadings of the 30 items on the single factor. All items demonstrated loadings above 0.40, with the majority exceeding 0.60, confirming that each item contributed meaningfully to the underlying construct.

**Table 2:** The results of descriptive statistics, internal consistencies, Geomin rotated loadings and Rasch analysis of AFTSS in EFA (Study 1).

Item	*M*	*SD*	Ske	Kur	geomin rotated loading	Rasch analysis 30 items			Rasch analysis 27 items			Rasch analysis 26 items		
						Infit	Outfit	PMC	Infit	Outfit	PMC	Infit	Outfit	PMC
Item 1	3.743	0.934	−0.714	0.601	0.416	1.993	2.793	0.466	/	/	/	/	/	/
Item 6	4.090	0.914	−0.990	0.771	0.626	1.588	1.597	0.645	/	/	/	/	/	/
Item 11	4.163	0.843	−1.062	1.456	0.767	1.037	0.986	0.747	1.148	1.128	0.741	1.168	1.137	0.744
Item 16	4.367	0.733	−1.329	2.904	0.778	0.991	0.801	0.718	1.056	0.885	0.718	1.056	0.886	0.724
Item 21	4.118	0.834	−1.124	2.019	0.761	0.981	0.879	0.754	1.105	1.083	0.743	1.131	1.096	0.745
Item 26	4.269	0.678	−0.788	1.489	0.860	0.518	0.480	0.816	0.563	0.535	0.810	0.575	0.541	0.813
Item 2	4.229	0.803	−1.061	1.470	0.684	1.239	1.223	0.673	1.410	1.551	0.664	1.451	1.673	0.662
Item 7	4.257	0.727	−0.760	0.722	0.660	1.124	1.098	0.675	1.266	1.261	0.672	1.300	1.269	0.674
Item 12	4.237	0.742	−0.836	0.962	0.774	0.883	0.800	0.751	0.947	0.937	0.752	0.963	0.937	0.755
Item 17	4.318	0.699	−1.038	2.002	0.748	0.901	0.731	0.726	0.968	0.798	0.730	0.979	0.808	0.734
Item 22	4.200	0.761	−1.086	2.146	0.842	0.682	0.567	0.804	0.713	0.612	0.803	0.721	0.619	0.806
Item 27	4.086	0.771	−0.851	1.719	0.656	1.109	1.260	0.679	1.280	1.483	0.672	1.297	1.468	0.677
Item 3	4.106	0.772	−0.617	0.368	0.662	1.044	1.265	0.676	1.199	1.563	0.669	1.257	1.658	0.663
Item 8	4.131	0.809	−0.804	0.580	0.696	1.114	1.360	0.696	1.242	1.492	0.695	1.272	1.494	0.698
Item 13	4.302	0.658	−0.761	1.523	0.758	0.764	0.661	0.743	0.833	0.744	0.745	0.850	0.753	0.748
Item 18	3.841	0.929	−0.666	0.376	0.520	1.759	2.106	0.557	/	/	/	/	/	/
Item 23	4.261	0.739	−1.073	2.197	0.824	0.743	0.623	0.785	0.779	0.675	0.786	0.783	0.671	0.791
Item 28	4.237	0.672	−0.647	1.142	0.835	0.547	0.495	0.813	0.612	0.565	0.805	0.623	0.566	0.809
Item 4	4.233	0.783	−1.105	1.840	0.616	1.383	1.373	0.628	1.563	1.662	0.624	/	/	/
Item 9	3.886	0.929	−0.727	0.479	0.697	1.154	1.284	0.708	1.305	1.485	0.702	1.334	1.493	0.704
Item 14	4.229	0.761	−1.143	2.587	0.840	0.688	0.582	0.800	0.742	0.642	0.796	0.759	0.650	0.798
Item 19	4.167	0.779	−0.930	1.444	0.833	0.712	0.635	0.801	0.777	0.715	0.795	0.797	0.730	0.796
Item 24	4.180	0.758	−0.882	1.185	0.816	0.705	0.640	0.798	0.784	0.733	0.789	0.794	0.742	0.792
Item 29	4.118	0.745	−0.734	0.968	0.739	0.886	1.029	0.725	0.969	1.084	0.730	0.989	1.088	0.733
Item 5	4.016	0.825	−0.826	1.150	0.636	1.205	1.384	0.656	1.359	1.619	0.654	1.425	1.739	0.646
Item 10	4.253	0.747	−1.044	1.988	0.748	0.943	0.964	0.732	1.010	0.990	0.736	1.026	0.980	0.741
Item 15	4.302	0.712	−1.132	2.714	0.880	0.557	0.450	0.819	0.566	0.465	0.819	0.576	0.471	0.821
Item 20	4.102	0.846	−1.016	1.390	0.742	1.083	1.030	0.727	1.181	1.236	0.723	1.204	1.248	0.726
Item 25	4.273	0.686	−1.027	2.892	0.842	0.605	0.507	0.795	0.625	0.530	0.800	0.637	0.533	0.803
Item 30	4.355	0.647	−0.866	1.867	0.789	0.692	0.577	0.760	0.742	0.628	0.761	0.757	0.655	0.763

Ske, Skewness; Kur, Kurtosis; Infit, information-weighted mean-square fit statistic; Outfit, outlier-sensitive mean-square fit statistic. Values close to 1.0 indicate good fit to the Rasch model. Values between 0.5 and 1.5 are considered acceptable. PMC, Point-Measure Correlation.

### Rasch model item purification

In the initial 30-item model, three items demonstrated misfit: Item 1 (infit = 1.99, outfit = 2.79, PMC = 0.47), Item 6 (infit = 1.59, outfit = 1.60, PMC = 0.65), Item 18 (infit = 1.76, outfit = 2.11, PMC = 0.56). We removed these three items, leaving 27 items for the second round.

In the second Rasch analysis with 27 items, Item 4 showed marginal misfit (infit = 1.56, outfit = 1.66, PMC = 0.62). Two further items showed outfit values slightly above 1.5, although their infit and point-measure correlations remained acceptable. We retained these items for further examination and proceeded to a third round of analysis. In the third round, we re-examined the 27-item model. Item 4 continued to show marginal fit and contributed limited unique information compared with other items, with its point-measure correlation (0.62) falling below the average of the remaining items. We therefore removed Item 4, resulting in a final 26-item scale. The final 26-item model demonstrated satisfactory infit across all retained items, with infit values ranging from 0.58 to 1.45 and point-measure correlations ranging from 0.65 to 0.82. Outfit values ranged from 0.54 to 1.74, with two items (Item 3 and Item 5) slightly above the 1.5 criterion. Because outfit is sensitive to isolated extreme responses, whereas infit and point-measure correlations were acceptable for these items, and because both items contributed to content coverage, they were retained. Sensitivity analyses excluding these items did not change the substantive conclusions.

### Unidimensionality and measurement precision

We examined the unidimensionality assumption of the final 26-item scale using principal component analysis of the standardized residuals from the Rasch model. The raw variance explained by the measures was 52.3%, exceeding the recommended threshold of 50%. The first contrast of the standardized residuals had an eigenvalue of 1.8, below the recommended criterion of 2.0. These results supported the unidimensionality of the 26-item AFTSS.

We also evaluated the measurement precision of the scale. The person reliability estimate from the Rasch analysis was 0.89, and the Cronbach’s alpha coefficient was 0.91, both indicating high internal consistency. The item separation index was 3.02 with an item reliability of 0.90, indicating that the items were spread across a sufficiently wide range of the satisfaction continuum and could reliably differentiate respondents with varying levels of satisfaction.

The Andrich thresholds for the five response categories were −2.14, −2.06, 0.19, and 4.01 logits. The ordered thresholds indicate that the five-point rating scale functioned as intended, with each category representing a distinct level of the latent trait. Category use was distributed across all five options, although Categories 4 and 5 together accounted for 85% of responses, reflecting the negatively skewed satisfaction scores in this sample.

### Preliminary differential item functioning analysis

A preliminary differential item functioning (DIF) analysis was conducted to examine whether the 26 items operated differently across gender and age groups. For gender comparisons, the sample was divided into male (*n* = 206) and female (*n* = 39) subgroups. DIF contrast values across all items ranged from −1.11 to 0.75 logits. For age comparisons, the sample was split into younger (age ≤ 22, *n* = 189) and older (age ≥ 23, *n* = 56) cohorts, with DIF contrast values ranging from −0.64 to 0.61 logits. Most DIF contrast values fell below the 0.64 logit criterion. However, three gender comparisons (Item 3, Item 8, Item 16) and one age comparison (Item 22) exceeded this threshold. Rasch-Welch tests indicated that these contrasts were statistically significant (*p* = 0.037, *p* = 0.002, *p* = 0.004, and *p* = 0.047, respectively). Given the small female and older-age subgroups, these results should be interpreted as preliminary evidence of possible DIF rather than as definitive measurement bias. Table 3 presents the complete DIF results for all 26 items across gender and age cohorts.

**Table 3:** Differential item functioning results across gender and age cohorts (Study 1).

Item	Gender DIF			Age DIF		
	Male	Female	DIF contrast	Younger age cohort (age ≤ 22)	Older adult age cohort (age ≥ 23)	DIF contrast
Item 11	0.210	−0.020	0.230	0.270	−0.240	0.510
Item 16	−0.970	0.080	−1.050	−0.650	−1.270	0.610
Item 21	0.430	−0.020	0.450	0.410	0.110	0.310
Item 26	−0.290	−0.230	−0.070	−0.240	−0.530	0.290
Item 2	−0.090	−0.230	0.140	−0.170	0.110	−0.280
Item 7	−0.200	−0.440	0.240	−0.240	−0.240	<0.001
Item 12	−0.250	0.270	−0.520	−0.260	0.270	−0.540
Item 17	−0.740	0.370	−1.110	−0.550	−0.430	−0.120
Item 22	−0.070	0.370	−0.430	−0.130	0.510	−0.640
Item 27	0.530	0.270	0.250	0.450	0.590	−0.130
Item 3	0.530	−0.230	0.750	0.290	0.810	−0.520
Item 8	0.410	−0.230	0.640	0.310	0.270	0.030
Item 13	−0.530	−0.120	−0.400	−0.450	−0.530	0.080
Item 23	−0.260	−0.330	0.080	−0.260	−0.340	0.080
Item 28	−0.180	−0.020	−0.160	−0.100	−0.340	0.230
Item 9	1.260	0.890	0.370	1.300	0.740	0.560
Item 14	−0.130	−0.020	−0.110	−0.030	−0.430	0.400
Item 19	0.160	0.180	−0.020	0.050	0.510	−0.460
Item 24	0.100	0.180	−0.070	0.010	0.430	−0.420
Item 29	0.330	0.460	−0.130	0.360	0.270	0.080
Item 5	0.860	0.180	0.680	0.750	0.660	0.080
Item 10	−0.220	−0.220	<0.001	−0.240	−0.150	−0.090
Item 15	−0.450	−0.450	<0.001	−0.500	−0.240	−0.260
Item 20	0.510	−0.020	0.530	0.390	0.510	−0.120
Item 25	−0.360	−0.120	−0.240	−0.310	−0.340	0.020
Item 30	−0.790	−0.330	−0.460	−0.630	−1.040	0.420

DIF, Differential Item Functioning. Male subgroup: *n* = 206; Female subgroup: *n* = 39. Younger age cohort (age ≤ 22 years, *n* = 189); older adult cohort (age ≥ 23 years, *n* = 56). DIF contrast values above 0.64 logits were considered meaningful. Significance was evaluated using the Rasch-Welch statistic.

### Short-form derivation

After establishing the psychometric properties of the 26-item full-length AFTSS, a 10-item short form was derived for applications requiring brief assessment. Item selection proceeded according to three criteria. The first criterion involved inspection of the item-person map (Wright map; see Figure 1) to identify items that targeted the range of the satisfaction continuum. The second criterion prioritized items with infit and outfit statistics closest to 1.0, indicating optimal fit to the Rasch model. The third criterion ensured that the selected items collectively covered the five content domains of the amateur football tournament experience, with two items retained from each domain to maintain content representativeness (Ziegler et al., 2014).

**Figure 1:**
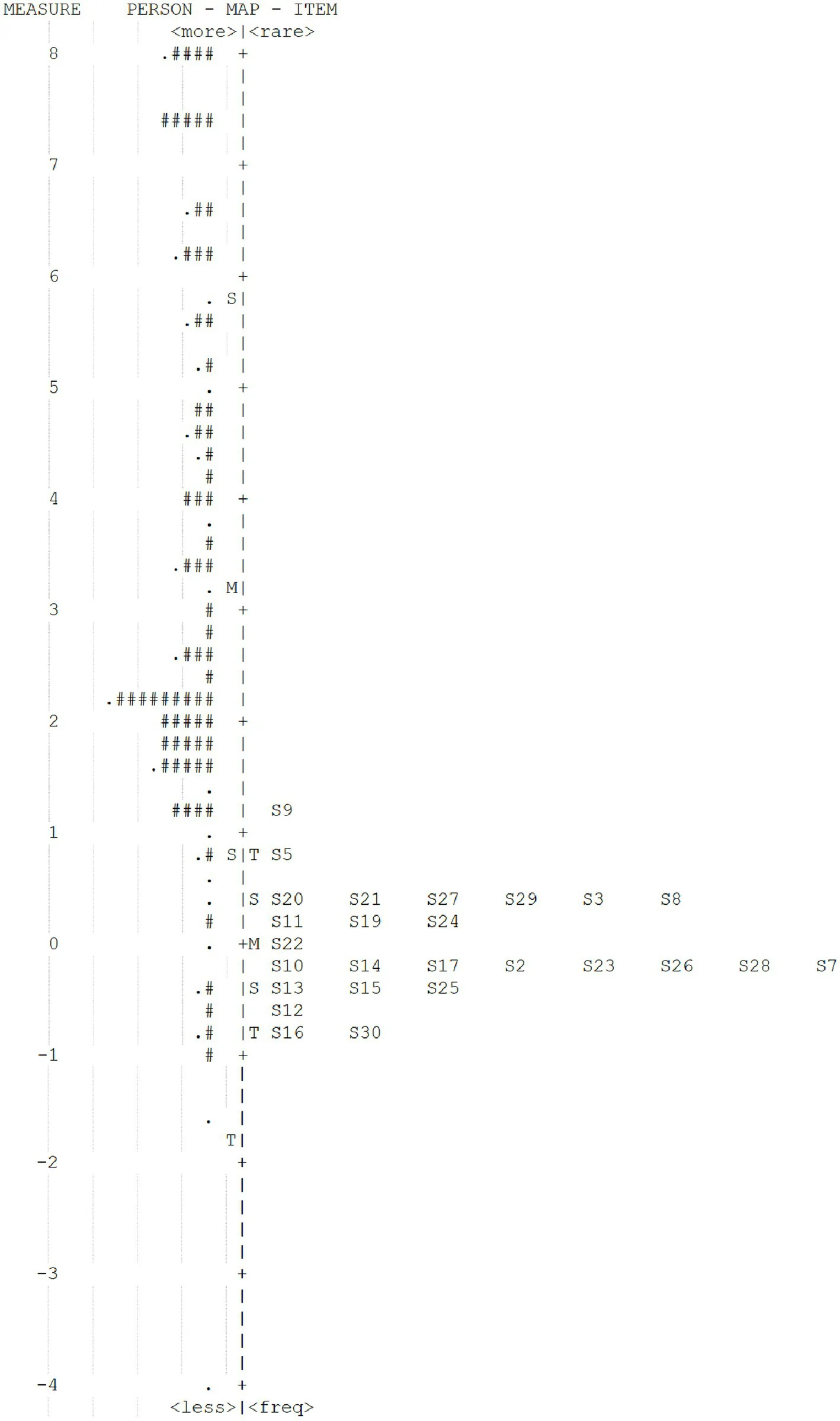
Wright map for the 10-item AFTSS-SF in Study 2.

Application of these criteria yielded 10 items from the final 26-item pool. The selected items demonstrated excellent Rasch fit, with infit values ranging from 0.58 to 0.99 and outfit values ranging from 0.53 to 0.99. The correlation between the total scores from the 26-item full-length scale and the 10-item short form was then computed within the same Study 1 sample. The two scores correlated at 0.974 (*p* < 0.001), which indicates substantial score correspondence between the two forms. It does not establish equivalent measurement precision because the short-form items were a subset of the full-length form and both forms were completed on the same occasion. Independent comparisons of person separation, reliability, test information, and conditional standard errors are needed.

## Discussion

In Study 1, we developed an initial 30-item pool for the Amateur Football Tournament Satisfaction Scale and purified it through exploratory factor analysis and Rasch model analyses. The final 26-item scale demonstrated satisfactory psychometric properties, including a unidimensional factor structure, acceptable item fit across three rounds of purification, and strong internal consistency.

The unidimensionality finding warrants particular attention. Although we generated items from five content domains of the amateur football experience, the Rasch residual principal component analysis supported a single underlying construct. This aligns with the leisure satisfaction framework, which conceptualizes satisfaction as a global evaluative judgment rather than a collection of independent subdimensions. This result justified treating tournament satisfaction as a unidimensional construct for subsequent analyses.

Sample size limitations in scale development are widely recognized (Morgado et al., 2018), and they are particularly consequential for DIF estimation, where subgroup sizes determine the stability of contrast estimates. The purification process removed four items that failed to meet Rasch fit criteria. These items primarily addressed tangible environmental features and administrative details. The preliminary DIF analysis suggested that most retained items functioned equivalently across gender and age groups. However, a small number of items showed statistically significant DIF, particularly in the gender comparison. These findings are preliminary because the female and older-age subgroups were small, and the items should be re-examined in a larger and more balanced sample.

The 26-item AFTSS offers a psychometrically sound foundation for measuring amateur football tournament satisfaction. In Study 2, we will use an independent sample to derive and validate a 10-item short form, examine criterion-related validity against established measures, and provide more robust evidence on measurement fairness.

## Study 2: confirmatory factor analysis and validation of the AFTSS short form

Study 2 aimed to cross-validate the 10-item short form of the Amateur Football Tournament Satisfaction Scale (AFTSS-SF) using an independent sample. The short form was derived from the 26-item full-length version based on Rasch fit statistics, item-person map inspection, and content representativeness across the five domains. Administration of the short form to a new sample of football major students from six sport universities in China provided the data for this validation. Confirmatory factor analysis (CFA) supported the unidimensional structure, while correlations with training satisfaction, athlete burnout, and group cohesion established criterion-related validity. The overarching goal was to establish a brief yet psychometrically robust instrument suitable for routine tournament evaluation and large-scale survey applications.

### Methods

#### Sample

Participants were recruited from six sport universities across China between July 2026. Using convenience sampling, we approached football major students enrolled in these institutions through university course coordinators and football programme directors, who distributed the survey link via institutional learning platforms and departmental social media groups. We selected this population because sport university students constitute a primary recruitment pool for amateur league competitions, giving them direct and regular exposure to organized tournament play.

A total of 856 major football students completed the initial survey. We applied two exclusion criteria: (a) excluding 99 participants who failed instructed response items embedded in the questionnaire; and (b) excluding 350 participants who showed invariant response patterns, defined as selecting the same response option across all items in a scale. We did not exclude participants based on prior amateur league participation because sport university students represent a key segment of the amateur football population regardless of formal league history, and we planned to conduct group comparisons between those with and without league experience. These screening criteria yielded a final analytic sample of 407 participants.

The final sample comprised 273 male participants and 134 female participants, with ages ranging from 18 to 25 years (*M* = 21.05, *SD* = 2.00). Participants reported an average of 8.5 years of formal football training (*SD* = 3.2). Regarding amateur league participation history, 192 participants reported prior experience in organized amateur leagues, and 215 reported no such experience.

### Measures

#### Amateur Football Tournament Satisfaction Scale Short Form

We administered the 10-item short form of the AFTSS derived from Study 1. The short form covers five content domains of amateur football tournament experience: venue environment, affective enjoyment, social identity and belonging, tournament organization, and personal development, with two items representing each domain. Each item is rated on a 5-point Likert scale ranging from 1 (strongly disagree) to 5 (strongly agree). We used the total score as the indicator of overall tournament satisfaction for all analysis. The short form demonstrated high internal consistency in this sample (Cronbach’s *α* = 0.897).

#### Training and competition satisfaction scale

We measured participants’ satisfaction with their training and competition experiences using a 6-item scale (Zhang and Liang, 2002). This instrument captures athletes’ positive evaluations of their preparation, performance, and competitive outcomes. Participants rated each item on a 5-point Likert scale ranging from 1 (strongly disagree) to 5 (strongly agree). We used this measure to examine convergent validity, expecting it to correlate positively with the AFTSS. The scale showed acceptable internal consistency in this sample (Cronbach’s *α* = 0.702).

#### Athlete burnout questionnaire

We measured athlete burnout using the 15-item athlete burnout questionnaire (Raedeke and Smith, 2001), which consists of three subscales that reduced sense of accomplishment (5 items), emotional and physical exhaustion (5 items), and devaluation of sport (5 items). Participants rated each item on a 5-point Likert scale ranging from 1 (almost never) to 5 (almost always). We used this measure to examine discriminant validity, expecting all three burnout dimensions to correlate negatively with AFTSS. In this sample, the emotional and physical exhaustion subscale (Cronbach’s α = 0.826) and the devaluation of sport subscale (Cronbach’s α = 0.820) demonstrated acceptable internal consistency, whereas the reduced sense of accomplishment subscale showed poor internal consistency (Cronbach’s α = 0.553).

#### Group environment questionnaire

We measured team cohesion using the 15-item group environment questionnaire (Carron et al., 1985), which consists of attraction to group-social (4 items), attraction to group-task (4 items), group integration-social (4 items), and group integration-task (3 items). Participants rated each item on a 5-point Likert scale ranging from 1 (strongly disagree) to 5 (strongly agree). Three of the four subscales demonstrated acceptable internal consistency in this sample, with Cronbach’s *α* = 0.873 for attraction to group-social, *α* = 0.812 for attraction to group-task, and *α* = 0.898 for group integration-task. However, the group integration-social subscale showed poor internal consistency (Cronbach’s *α* = 0.244). Given that this value fell well below the acceptable threshold, we excluded this subscale from subsequent validity analyses.

#### Demographic and football experience variables

Demographic information was collected, including age and gender. Participants reported their gender (1 = male, 2 = female) and age in years. Prior experience participating in organized amateur leagues was also assessed, with examples provided including corporate leagues, university-level competitions, provincial super leagues, and municipal super leagues (1 = yes, 2 = no).

### Procedure

The survey was administered online through a widely used Chinese survey platform (*Wenjuanxing*). The questionnaire opened with an informed consent statement that explained the purpose of the study, the voluntary nature of participation, and the confidentiality of all responses. Participants provided electronic consent by clicking “I agree to participate” before accessing the questionnaire items. The survey contained the 10-item short form of the AFTSS derived from Study 1, three criterion measures for validity testing, and demographic questions. The survey was designed to take approximately 10 to 12 min to complete. No monetary compensation or course credit was offered for participation. The research protocol received ethical approval from the institutional review board of the corresponding author’s university (IRB: 2026LCLL-097).

### Data analysis

All analyses were conducted using SPSS 26.0, Mplus 8.4, and WINSTEPS 5.6.0.0. Descriptive statistics including means, standard deviations, skewness, and kurtosis were calculated for all study variables. Pearson correlations were then computed among the AFTSS short form total score, the training and competition satisfaction scale, the three burnout subscales, and the three retained group cohesion subscales to examine the pattern of relationships between tournament satisfaction and the criterion measures.

A confirmatory factor analysis was performed on the 10-item short form to verify the unidimensional factor structure identified in Study 1. Model fit was evaluated using the comparative fit index, the Tucker-Lewis index, the root mean square error of approximation, and the standardized root mean square residual. Values of 0.90 or higher for CFI and TLI, 0.08 or lower for RMSEA, and 0.08 or lower for SRMR were considered indicative of acceptable fit. Composite reliability and average variance extracted were also calculated to assess the internal consistency and convergent validity of the short form.

To examine criterion-related validity, we correlated the AFTSS short form total score with the training and competition satisfaction scale, the three burnout subscales, and the three retained group cohesion subscales. Positive correlations were expected with training and competition satisfaction and with the retained group cohesion dimensions, whereas negative correlations were expected with the burnout dimensions.

Rasch-based differential item functioning analysis was conducted using the Mantel–Haenszel method to examine whether the 10 items operated differently across gender and league participation groups (Meade and Lautenschlager, 2004). Unlike the preliminary DIF analysis in Study 1, which was limited by imbalanced subgroups, the Study 2 sample provided sufficient statistical power for stable DIF estimation, with male and female subgroups each exceeding 100 participants. DIF contrast values above 0.64 logits were considered indicative of meaningful differential item functioning.

## Results

### Descriptive statistics and correlations

Table 4 displays the means, standard deviations, skewness, and kurtosis for the AFTSS short form total score, the training and competition satisfaction scale, the three burnout subscales, and the three retained group cohesion subscales. The AFTSS short form total score showed a mean of 4.01 (*SD* = 0.61), with skewness of −0.66 and kurtosis of 0.81, indicating acceptable univariate normality.

**Table 4:** Descriptive statistics and correlations (Study 2).

No.	Variables	*M*	*SD*	Ske	Kur	ɑ	ω	1	2	3	4	5	6	7	8	9	10
1	Amateur football tournament satisfaction	4.012	0.612	−0.657	0.807	0.897	0.905										
2	Training and competition satisfaction	5.179	0.840	−0.336	0.377	0.702	0.849	0.648^***^									
3	Reduced sense of accomplishment	2.462	0.591	−0.148	−0.628	0.553	0.590	−0.494^***^	−0.486^***^								
4	Emotional and physical exhaustion	2.565	0.800	0.512	0.27	0.826	0.833	−0.262^***^	−0.255^***^	0.647^***^							
5	Devaluation of sport	2.530	0.863	0.388	0.041	0.820	0.828	−0.273^***^	−0.256^***^	0.721^***^	0.817^***^						
6	Attraction to group – social	5.773	0.998	−0.58	0.101	0.873	0.878	0.534^***^	0.501^***^	−0.532^***^	−0.326^***^	−0.324^***^					
7	Attraction to group – task	5.704	1.050	−0.575	−0.137	0.812	0.825	0.537^***^	0.527^***^	−0.525^***^	−0.332^***^	−0.364^***^	0.878^***^				
8	Group integration – social	4.559	0.847	0.846	0.713	0.244	0.475	0.185^**^	0.180^**^	0.097	0.177^**^	0.257^***^	0.218^***^	0.233^***^			
9	Group integration – task	5.746	0.986	−0.268	−1.021	0.898	0.906	0.557^***^	0.486^***^	−0.498^***^	−0.338^***^	−0.325^***^	0.778^***^	0.796^***^	0.274^***^		
10	Gender	/	/	/	/	/	/	−0.175^***^	−0.201^***^	0.089	0.066	0.034	−0.191^***^	−0.220^***^	−0.136^*^	−0.209^***^	
11	Age	21.050	1.996	/	/	/	/	0.119^*^	0.095	−0.069	−0.033	−0.031	0.114^*^	0.122^*^	0.044	0.121^*^	−0.209^***^

^*^p < 0.05, ^**^p < 0.01, ^***^p < 0.001. Ske., Skewness; Kur., Kurtosis.

Pearson correlations among all study variables are also presented in Table 4. The AFTSS short form correlated positively with training and competition satisfaction (*r* = 0.648, *p* < 0.001) and negatively with all three burnout subscales, with the strongest negative correlation emerging for reduced sense of accomplishment (*r* = −0.494, *p* < 0.001), followed by devaluation of sport (*r* = −0.273, *p* < 0.001) and emotional and physical exhaustion (*r* = −0.262, *p* < 0.001). Positive correlations also emerged between the AFTSS short form and the retained group cohesion subscales: attraction to group-social (*r* = 0.534, *p* < 0.001), attraction to group-task (*r* = 0.537, *p* < 0.001), and group integration-task (*r* = 0.557, *p* < 0.001). These correlations all aligned with theoretical expectations and provided initial support for the criterion-related validity of the AFTSS short form.

### Confirmatory factor analysis

Confirmatory factor analysis on the 10-item AFTSS short form verified the unidimensional factor structure identified in Study 1. The model demonstrated acceptable fit to the data: χ^2^(35) = 83.21, CFI = 0.962, TLI = 0.951, RMSEA = 0.058, SRMR = 0.039. All factor loadings reached statistical significance and ranged from 0.423 to 0.931. Composite reliability for the scale was 0.921, exceeding the recommended threshold of 0.70, and average variance extracted was 0.612, exceeding the recommended threshold of 0.50. Together, these results supported the unidimensional structure and convergent validity of the 10-item short form. Table 5 presents the factor loadings for all 10 items.

**Table 5:** Factor loadings of the Amateur Football Tournament Satisfaction Scale Short Form (AFTSS-SF).

Item	Content	Factor loading	*SE*	*p*
1	在比赛中, 我能按照自己的意愿自由发挥 I am free to play to my full potential during competitions.	0.423	0.043	<0.001
2	赛事的场地设施 (如更衣室, 饮水点, 医疗点) 是完善的 Supporting facilities (changing rooms, drinking stations, medical booths) are fully equipped.	0.520	0.035	<0.001
3	无论比赛结果如何, 我仍为能参与这场比赛感到自豪 I feel proud to compete regardless of the final match result.	0.787	0.022	<0.001
4	我与对手之间的交流是友好且尊重的 My communications with opponents are friendly and respectful.	0.798	0.021	<0.001
5	观众的热情和助威声让我更有比赛动力 The enthusiasm and cheers from spectators raise my drive to compete.	0.795	0.021	<0.001
6	这场比赛让我暂时忘记了生活中的压力 Playing in this competition allows me to temporarily escape daily stress.	0.808	0.019	<0.001
7	赛场的光线、温度、通风等环境条件让我感到舒适 Environmental factors such as lighting, temperature and ventilation at the venue are comfortable.	0.861	0.014	<0.001
8	我感受到自己属于这支球队和这次赛事的一部分 I have a strong sense of belonging to my team and this competition.	0.931	0.009	<0.001
9	赛程安排合理, 不会让我感到过度疲劳 The competition schedule is reasonably arranged without excessive fatigue.	0.752	0.022	<0.001
10	我认为这次赛事对我的成长是有帮助的 This competition contributes to my long-term athletic development.	0.868	0.016	<0.001

### Rasch model fit for the short form

Fitting the 10-item short form to the Rasch rating scale model allowed us to examine item-level fit statistics and differential item functioning. Infit statistics ranged from 0.58 to 1.60, and outfit statistics ranged from 0.53 to 1.76. Most items fell within the acceptable 0.5 to 1.5 range. Item 2 showed slightly elevated values (infit = 1.60, outfit = 1.66) that approached but did not substantially exceed the threshold. Given that point-measure correlations for all items ranged from 0.48 to 0.83, we retained all 10 items for the short form. Item 1 also showed an elevated outfit value (1.76) in Table 6, whereas its infit and point-measure correlation were acceptable. The Andrich thresholds for the short form were −2.08, −1.88, 0.74, and 3.21 logits, again indicating ordered and functioning response categories. For the 10-item short form, the item separation index was 7.50 with an item reliability of 0.98, indicating that the short-form items were well separated along the satisfaction continuum. The person-item distribution for the 10-item short form is presented in Figure 2. Local dependence among the five content pairs was examined using residual correlations from the Rasch model. All paired residual correlations fell below the |0.20| threshold, indicating no substantial local dependence. An additional interpretive consideration concerns Item 8, which combines team belonging and event belonging within a single statement. This item showed a strong factor loading and favorable Rasch fit in the present sample, suggesting that the dual referent did not materially distort responses. Future research could examine whether separating the two referents improves interpretability.

**Table 6:** Infit, outfit, PMC and differential item functioning results for short-form AFTSS items (Study 2).

Item	Infit	Outfit	PMC	Gender DIF			League Experience DIF		
				Male	Female	DIF contrast	With league participation	Without league participation	DIF contrast
Item 1	1.502	1.756	0.484	0.970	0.970	<0.001	1.120	0.680	0.440
Item 2	1.599	1.658	0.599	1.240	1.530	−0.290	1.560	1.090	0.480
Item 3	0.891	0.901	0.750	0.090	−0.060	0.150	0.040	−0.050	0.080
Item 4	0.929	0.846	0.729	−0.370	−0.600	0.230	−0.350	−0.760	0.410
Item 5	1.065	0.935	0.719	−0.820	−0.530	−0.290	−0.740	−0.500	−0.240
Item 6	0.838	0.937	0.724	−0.590	−0.610	0.020	−0.750	−0.320	−0.430
Item 7	0.792	0.785	0.793	0.140	<0.001	0.140	−0.110	0.370	−0.480
Item 8	0.576	0.531	0.830	−0.390	−0.350	−0.030	−0.440	−0.210	−0.220
Item 9	1.020	1.001	0.730	0.460	0.170	0.290	0.300	0.300	<0.001
Item 10	0.742	0.726	0.773	−0.800	−0.570	−0.230	−0.750	−0.520	−0.230

Infit, information-weighted mean-square fit statistic; Outfit, outlier-sensitive mean-square fit statistic. Values close to 1.0 indicate good fit to the Rasch model. Values between 0.5 and 1.5 are considered acceptable. PMC, Point-Measure Correlation; DIF, Differential Item Functioning. Male subgroup: *n* = 273; Female subgroup: *n* = 134. League participation refers to participation in formal municipal/provincial recreational football leagues.

**Figure 2:**
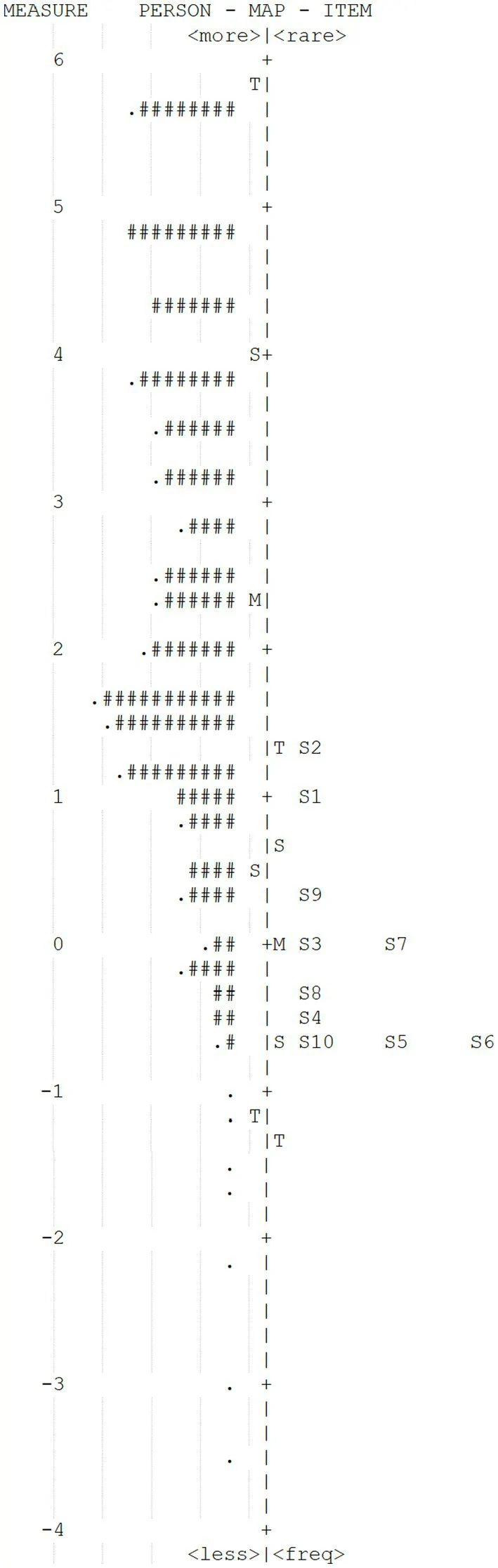
Wright map for the 26-item AFTSS in Study 1.

### Criterion-related validity

To examine criterion-related validity, we correlated the AFTSS-SF total score with the theoretically related constructs. The strong positive correlation with training and competition satisfaction (*r* = 0.648, *p* < 0.001) supported convergent validity, as did the positive correlations with the three retained group cohesion subscales. The negative correlations with burnout subscales provided evidence for discriminant validity, with the strongest negative correlation again appearing for reduced sense of accomplishment (*r* = −0.494, *p* < 0.001). These results supported that the AFTSS-SF related to external criteria in theoretically expected ways. Table 4 presents the full correlation matrix including all criterion variables.

### Differential item functioning

Rasch-based DIF analysis using the Mantel–Haenszel method examined whether the 10 items operated differently across gender and league participation groups. For gender, DIF contrast values ranged from −0.29 to 0.29 logits, all falling below the conventional 0.64 logit threshold and indicating no substantial gender-related differential functioning. For league participation experience, DIF contrast values ranged from −0.48 to 0.48 logits, again all below the 0.64 threshold. Table 6 presents the DIF contrast values for each item across gender and league participation groups. These results provide evidence for measurement fairness across these subgroups based on DIF analysis.

## Discussion

In Study 2, we independently validated the 10-item short form of the Amateur Football Tournament Satisfaction Scale (AFTSS-SF) using a new sample of 407 major football students. Confirmatory factor analysis supported the unidimensional structure, with all factor loadings exceeding 0.40. The scale demonstrated high internal consistency and composite reliability, and the pattern of correlations with criterion measures aligned with theoretical expectations.

The criterion-related validity findings showed that the AFTSS-SF correlated most strongly with training and competition satisfaction and with the retained group cohesion subscales, while showing negative correlations with burnout dimensions, particularly reduced sense of accomplishment (Strauss and Smith, 2009). This differential pattern is consistent with prior research distinguishing between cognitive and affective components of athlete burnout.

Rasch-based differential item functioning analysis revealed that all 10 items functioned equivalently across gender and league participation groups, with all DIF contrast values falling below the 0.64 logit threshold. This finding provides evidence for the measurement fairness of the short form across these subgroups.

Together with Study 1, the present findings provide support for the AFTSS-SF as a psychometrically sound instrument for measuring amateur football tournament satisfaction in both research and applied settings. We now turn to the general discussion to synthesize findings across both studies and address broader implications.

## General discussion

This study developed and validated the Amateur Football Tournament Satisfaction Scale (AFTSS) as the first dedicated instrument for measuring satisfaction specifically in amateur football tournament contexts. Across two independent samples totaling over 800 participants, we established a 26-item full-length version and a 10-item short form, both demonstrating strong psychometric properties. The findings support a unidimensional factor structure, satisfactory reliability, criterion-related validity, and preliminary measurement fairness across gender and league participation groups. These results not only fill a gap in sport satisfaction measurement but also offer empirical support for the application of Rasch methodology in amateur sport research.

### Extending prior work on sport satisfaction measurement

The present findings align with and extend existing measurement work in sport and leisure satisfaction. Our unidimensional conceptualization of tournament satisfaction resonates with the leisure satisfaction framework (Beard and Ragheb, 1980), which treats satisfaction as a global evaluative judgment formed from multiple experiential inputs. While Beard and Ragheb developed their framework in the context of general leisure activities, the present findings demonstrate that this global judgment mechanism operates similarly in the specific context of competitive amateur sport, a setting characterized by higher stakes, structured competition, and collective team dynamics. Like their foundational work, we found that diverse aspects of the tournament experience coalesce into a unified overall perception rather than operating as independent subdimensions.

This pattern also echoes the theoretical position of Riemer and Chelladurai (1998), who conceptualized athlete satisfaction as a multifaceted yet coherent construct in their development of the Athlete Satisfaction Questionnaire. Although the ASQ comprises 15 distinct satisfaction dimensions, its underlying structure reveals substantial inter-factor correlations that suggest a unifying higher-order factor. The present findings extend this observation by demonstrating that amateur players’ satisfaction judgments coalesce into a single overarching evaluation without the need for multiple subdimensions. This difference may reflect the distinct motivational orientations and experiential priorities of amateur versus elite populations.

The unidimensional structure observed in the present study further aligns with research in event satisfaction, where satisfaction with participation experiences has been operationalized as a global evaluative construct (Sato et al., 2017). Collectively, these convergent findings suggest that while the content and sources of satisfaction may differ across populations, the fundamental organization of satisfaction as a global integrative judgment appears robust across leisure, amateur sport, and event participation contexts.

The pattern of criterion correlations we observed further parallels established relationships in the sport satisfaction literature. The positive association between tournament satisfaction and training and competition satisfaction mirrors findings from previous work demonstrating that athletes’ satisfaction across different sport contexts tends to correlate positively (Gledhill et al., 2017; Scanlan et al., 1993). Likewise, the negative associations we observed between satisfaction and burnout dimensions, particularly reduced sense of accomplishment, are consistent with prior research documenting the protective function of positive sport experiences against athlete burnout (Raedeke and Smith, 2001). These convergent findings confirm that our scale captures theoretically expected associations and operates in ways consistent with established measurement tools (Flake et al., 2017).

### Addressing the three identified gaps

This study makes three contributions that directly address the research gaps identified in the Introduction. The first contribution is the development of the first football-specific tournament satisfaction instrument. The AFTSS fills this gap by providing items that reflect the unique characteristics of amateur football tournament participation, including venue environment, affective enjoyment, social identity, tournament organization, and personal development (Ivarsson et al., 2020). This football-specific focus is not merely a matter of content substitution, addressing a fundamental conceptual issue in satisfaction measurement. The complexity of football’s social meaning in China is further evidenced by public debates surrounding the naturalization of players, which have revealed diverse and contested understandings of national identity (Han, 2023).

The second contribution concerns the application of Rasch model analysis to provide item-level diagnostic evidence that classical test theory alone cannot offer. Most existing instruments in this field rely exclusively on classical test theory, producing sample-dependent estimates and lacking item-level fit information (Flake et al., 2017). The present Rasch-based approach allowed systematic identification and removal of misfitting items, rigorous testing of the unidimensionality assumption, and examination of differential item functioning across demographic groups. The interval-level measurement properties of the AFTSS distinguish it from ordinal raw scores produced by CTT instruments, making it suitable for parametric statistical models that assume normally distributed continuous variables. This approach parallels recent applications of Rasch methodology in sport psychology, where item-level diagnostic evidence has proven valuable for validating psychosocial measures in athletic populations (Hwang et al., 2025). Additionally, given that youth sport participation can be stratified along racial and socioeconomic lines (Fox and Paradies, 2020), the measurement fairness of the AFTSS across demographic subgroups represents a particularly important psychometric property.

The third contribution addresses the absence of paired full-length and short-form instruments in amateur sport satisfaction assessment. The 26-item full-length version provides comprehensive measurement for academic research requiring detailed content coverage and precision. The 10-item short form provides a more efficient assessment option and shows strong total-score correspondence with the full-length version. Evidence that the two forms have comparable measurement precision remains to be established. The high correlation between the two forms (*r* = 0.974) is substantial, but it partly reflects shared item content and same-occasion administration. Researchers should therefore treat the short form as a promising brief measure rather than a fully equivalent substitute for the full-length version until independent precision comparisons are available.

The high correlation between the two forms is substantial, but it partly reflects shared item content and same-occasion administration. This paired availability gives researchers and practitioners flexibility to select the instrument length appropriate to their specific assessment needs without sacrificing measurement quality. The development of paired full-length and short-form versions aligns with emerging practices in sport and exercise psychology measurement, where brief instruments have demonstrated comparable psychometric quality to their longer counterparts while substantially reducing respondent burden (Jo et al., 2026).

### Practical implications

The AFTSS full-length and short-form versions offer practical value for different stakeholders in amateur football. Tournament organizers can use the short form for routine satisfaction monitoring across seasons. With only 10 items, the short form can be administered at the end of each tournament or even at multiple points during a league season without overburdening players. The brief format directly addresses the low response rates that often characterise routine evaluation in amateur sport settings, where players have limited time and competing priorities. Organizers can aggregate satisfaction scores across matches to identify patterns, such as declining satisfaction related to specific venues, scheduling patterns, or officiating quality, and make targeted improvements based on empirical evidence. The unidimensional structure simplifies interpretation, as organizers can track a single overall satisfaction score rather than monitoring multiple subscales simultaneously.

For academic researchers investigating the antecedents or consequences of tournament satisfaction, the 26-item full-length version provides greater content coverage and measurement precision. Researchers can administer the full-length version when satisfaction serves as a primary outcome variable or when detailed comparisons across content domains are of theoretical interest. The interval-level measurement properties of the scale make it suitable for use in parametric statistical models that assume normally distributed continuous variables, a feature that distinguishes it from ordinal raw scores produced by many classical test theory instruments.

The differential correlation patterns observed between tournament satisfaction and burnout dimensions suggest practical intervention directions. The strongest negative association with reduced sense of accomplishment indicates that satisfaction most closely relates to players’ feelings of competence and achievement. Organizers seeking to protect players from burnout might prioritise elements that enhance feelings of accomplishment, such as providing meaningful competition, recognising player contributions, and ensuring balanced competition that allows all teams to experience success. Conversely, the weaker association with emotional and physical exhaustion suggests that tournament satisfaction may not directly address the physical demands of competition, which organizers should manage through scheduling and recovery support rather than relying on satisfaction enhancement alone.

### Limitations and future directions

Several limitations should be considered when interpreting our findings. First, both studies relied on self-reported data collected through online questionnaires. Although we excluded inattentive responders and those showing invariant response patterns, common method variance may still have inflated observed correlations among variables. Future research could incorporate objective indicators of tournament quality, such as facility ratings from independent assessors or behavioral measures of player retention, to complement self-reported satisfaction data.

Second, the Study 2 sample consisted of sport university students aged 18 to 25, which is narrower than the general amateur football population represented in Study 1 (aged 18 to 45). This age and population gap limits how far the short form’s validation can be generalized to the broader amateur football population. Future studies should validate the short form in more diverse amateur samples.

Third, our samples were drawn from Chinese amateur football contexts, including sport university students and participants in provincial, municipal, and commercial leagues. The cross-cultural applicability of the AFTSS requires empirical verification in other regions and football cultures. Researchers should translate the scale into other languages and examine its psychometric properties in diverse amateur football populations before making cross-cultural comparisons. The unidimensional structure and item functioning may differ across cultural contexts, and these possibilities warrant investigation.

Fourth, the study design was cross-sectional, which limits conclusions about causal relationships between tournament satisfaction and its correlates. Longitudinal studies tracking players across multiple tournaments could examine whether satisfaction changes over time and whether early satisfaction predicts subsequent participation behavior, such as league retention and tournament attendance. Such designs would provide stronger evidence for the predictive validity of the AFTSS.

Fifth, the data quality screening procedures resulted in a notable reduction in sample size, particularly in Study 1. While conservative screening enhanced data reliability (Huang et al., 2012), the generalisability of findings to less attentive or less experienced responders remains unknown. In addition, some respondents who selected the same response option across all items may have been highly satisfied and answering honestly rather than inattentive. Because these respondents were excluded before the main analysis, we could not directly compare their satisfaction scores with those of retained respondents. Future research could examine whether similar psychometric properties emerge with less restrictive exclusion criteria and could compare satisfaction scores between excluded and retained respondents. This would help determine whether the high exclusion rate reflects the effectiveness of the screening procedures or a potential source of selection bias.

Finally, the Group Environment Questionnaire’s group integration-social subscale showed poor internal consistency in our Study 2 sample and was excluded from analysis. In contrast, the Reduced Sense of Accomplishment subscale was retained despite low reliability because it represents a theoretically central dimension of athlete burnout and its correlation with tournament satisfaction was a key criterion-related validity finding. However, this low reliability is acknowledged as a limitation, and the magnitude of the correlation should be interpreted with caution. The group integration-social subscale was excluded because its reliability was substantially lower and it was not theoretically essential to the validity argument. Future research should examine the cross-cultural applicability of existing group cohesion measures in non-Western amateur sport settings and consider developing culturally adapted measures of social cohesion for Chinese amateur football populations.

## Conclusion

This study developed and validated the Amateur Football Tournament Satisfaction Scale as a dedicated measurement tool for amateur football tournament satisfaction. The full-length version provides 26 items with strong psychometric properties, and the short form offers 10 items with strong total-score correspondence to the full-length version. Independent evidence of comparable measurement precision is still needed. Both versions showed a unidimensional structure and satisfactory reliability, and the short form demonstrated criterion-related validity in Study 2. Preliminary DIF analyses offered partial support for measurement fairness, with most items functioning equivalently but a few Study 1 items showing statistically significant DIF that warrants further examination. Researchers and practitioners can select the version appropriate to their assessment needs, whether for academic research requiring detailed measurement or for routine tournament evaluation requiring efficiency. By providing two complementary instruments and establishing their psychometric properties through Rasch model analysis, this study offers a foundation for advancing research and practice in amateur football tournament satisfaction.

## Data Availability

The original contributions presented in the study are included in the article/Supplementary material, further inquiries can be directed to the corresponding author.

## References

[ref1] Armbrecht J. Andersson T. D. (2020). The event experience, hedonic and eudaimonic satisfaction and subjective well-being among sport event participants. J. Policy Res. Tour. Leis. Events 12, 457–477. doi: 10.1080/19407963.2019.1695346

[ref2] Beard J. G. Ragheb M. G. (1980). Measuring leisure satisfaction. J. Leis. Res. 12, 20–33. doi: 10.1080/00222216.1980.11969416

[ref3] Benson A. J. Bruner M. W. (2018). How teammate behaviors relate to athlete affect, cognition, and behaviors: a daily diary approach within youth sport. Psychol. Sport Exerc. 34, 119–127. doi: 10.1016/j.psychsport.2017.10.008

[ref4] Boateng G. O. Neilands T. B. Frongillo E. A. Melgar-Quiñonez H. R. Young S. L. (2018). Best practices for developing and validating scales for health, social, and behavioral research: a primer. Front. Public Health 6, 149–166. doi: 10.3389/fpubh.2018.00149, 29942800 PMC6004510

[ref5] Bond T. (2015). Applying the Rasch Model. Routledge.

[ref6] Carpenter S. (2018). Ten steps in scale development and reporting: a guide for researchers. Commun. Methods Meas. 12, 25–44. doi: 10.1080/19312458.2017.1396583

[ref7] Carron A. Widmeyer W. Brawley L. (1985). The development of an instrument to assess cohesion in sport teams—the group environment questionnaire. J. Sport Exerc. Psychol. 7, 244–266. doi: 10.1123/jsp.7.3.244

[ref8] Chen N. Cheng A. Zheng X. Liao X.-L. Li W. Chen I.-H. . (2026). Psychometric evaluation of the Chinese snyder dispositional hope scale and adaptability scale among Chinese EFL college students. Acta Psychol. 262:106028. doi: 10.1016/j.actpsy.2025.106028, 41338103

[ref9] Chen H. Dai J. Sun H. (2019). Validation of a Chinese version of the physical activity enjoyment scale: factorial validity, measurement equivalence, and predictive validity. Int. J. Sport Exerc. Psychol. 17, 367–380. doi: 10.1080/1612197X.2017.1354054

[ref10] Deci E. L. Ryan R. M. (2000). The “what” and “why” of goal pursuits: human needs and the self-determination of behavior. Psychol. Inq. 11, 227–268. doi: 10.1207/S15327965PLI1104_01

[ref11] Dong A. Zhang H. Kong L. Lu T. Zheng C. Ai F. . (2024). Chinese version of the physical resilience scale (PRS): Reliability and validity test based on Rasch analysis. BMC Public Health 24:2541. doi: 10.1186/s12889-024-19978-6, 39294642 PMC11411801

[ref12] Flake J. K. Pek J. Hehman E. (2017). Construct validation in social and personality research: current practice and recommendations. Soc. Psychol. Personal. Sci. 8, 370–378. doi: 10.1177/1948550617693063

[ref13] Fox B. Paradies Y. (2020). Youth sport and community segregation: a study of kids’ participation in Australian rules football and soccer clubs in an Australian community. Race Ethn. Educ. 23, 732–746. doi: 10.1080/13613324.2019.1679755

[ref14] Gledhill A. Harwood C. Forsdyke D. (2017). Psychosocial factors associated with talent development in football: a systematic review. Psychol. Sport Exerc. 31, 93–112. doi: 10.1016/j.psychsport.2017.04.002

[ref15] Han P. (2023). Can they represent the nation? Nationalism, national identity, and naturalized athletes in Chinese football. Natl. Identities 25, 339–355. doi: 10.1080/14608944.2022.2125943

[ref16] Hergesell A. (2022). Using Rasch analysis for scale development and refinement in tourism: theory and illustration. J. Bus. Res. 142, 551–561. doi: 10.1016/j.jbusres.2021.12.063

[ref17] Huang J. L. Curran P. G. Keeney J. Poposki E. M. DeShon R. P. (2012). Detecting and deterring insufficient effort responding to surveys. J. Bus. Psychol. 27, 99–114. doi: 10.1007/s10869-011-9231-8

[ref18] Hwang S. Ryu S. Lee S. Jang D. Ryu M.-A. (2025). Assessing implicit beliefs in elite young soccer players: a Rasch model analysis of the conceptions of the nature of athletic ability questionnaire-2. Meas. Phys. Educ. Exerc. Sci. 29, 443–453. doi: 10.1080/1091367X.2025.2497046

[ref19] Ivarsson A. Kilhage-Persson A. Martindale R. Priestley D. Huijgen B. Ardern C. . (2020). Psychological factors and future performance of football players: a systematic review with meta-analysis. J. Sci. Med. Sport 23, 415–420. doi: 10.1016/j.jsams.2019.10.021, 31753742

[ref20] Jiang K. Bairner A. (2020). Chinese football fandom and civic identities: a study of the fans of Shanghai Shenhua and Beijing Guoan. Sport Soc. 23, 2078–2098. doi: 10.1080/17430437.2020.1835859

[ref21] Jo E. Kim H.-R. Oh S.-E. Choi C.-H. (2026). Validation of a flow scale for online physical education lectures using rasch analysis. Meas. Phys. Educ. Exerc. Sci., 1–10. doi: 10.1080/1091367X.2026.2643885, 37339054

[ref22] Kim S.-H. Cho D. (2022). Psychometric properties of leisure satisfaction scale (LSS)-short form: a Rasch rating model calibration approach. BMC Psychol. 10:151. doi: 10.1186/s40359-022-00861-1, 35706062 PMC9202183

[ref23] Lambert L. S. Newman D. A. (2023). Construct development and validation in three practical steps: recommendations for reviewers, editors, and authors. Organ. Res. Methods 26, 574–607. doi: 10.1177/10944281221115374

[ref24] Lee C. W. (2022). Defending local culture through the global game in southern China: Guangzhou football club fan culture. Eur. J. Sport Soc. 19, 56–77. doi: 10.1080/16138171.2021.1878437

[ref25] Lianopoulos Y. Kotsi N. Karagiorgos T. Theodorakis N. D. (2024). Experiential effects on mass sport participants’ event satisfaction and behavioral intentions: examining sensory, affective, behavioral, intellectual and relational dimensions. Int J. Event Festiv. Manag. 15, 270–292. doi: 10.1108/IJEFM-09-2023-0075

[ref26] Magnusen M. Marsh P. Petersen J. (2023). The collegiate sport experience: reconceptualizing sport venues and college athlete satisfaction: reconceptualizing sport venues. J. Amateur Sport 9. doi: 10.17161/jas.v9i1.18398

[ref27] Meade A. W. Lautenschlager G. J. (2004). A comparison of item response theory and confirmatory factor analytic methodologies for establishing measurement equivalence/invariance. Organ. Res. Methods 7, 361–388. doi: 10.1177/1094428104268027

[ref28] Morgado F. F. R. Meireles J. F. F. Neves C. M. Amaral A. C. S. Ferreira M. E. C. (2018). Scale development: Ten main limitations and recommendations to improve future research practices. Psicol. Reflex. Crít. 30, 3–22. doi: 10.1186/s41155-016-0057-1, 32025957 PMC6966966

[ref29] Murero M. Rice R. E. (2013). The Internet and Health Care: Theory, Research, and Practice. New York, NY: Routledge.

[ref30] Öcal K. (2016). Tournament satisfaction scale (TOSS). J. Hum. Sci. 13:2186. doi: 10.14687/ijhs.v13i1.3781

[ref31] Pierobon A. Krägeloh C. (2026). Rasch analysis in the development of self-reported outcome measures to assess physical function. Musculoskelet. Sci. Pract. 83:103544. doi: 10.1016/j.msksp.2026.103544, 41856028

[ref32] Polit D. F. Beck C. T. (2006). The content validity index: are you sure you know what’s being reported? Critique and recommendations. Res. Nurs. Health 29, 489–497. doi: 10.1002/nur.20147 16977646

[ref33] Raedeke T. D. Smith A. L. (2001). Development and preliminary validation of an athlete burnout measure. J. Sport Exerc. Psychol. 23, 281–306. doi: 10.1123/jsep.23.4.281 28682196

[ref34] Riemer H. Chelladurai P. (1998). Development of the Athlete Satisfaction Questionnaire (ASQ). J. Sport Exerc. Psychol. 20, 127–156. doi: 10.1123/jsep.20.2.127

[ref35] Rodrigues F. Monteiro D. Matos R. Jacinto M. Antunes R. Amaro N. (2024). Exploring the dynamics of athletes’ enjoyment and self-determined motivation, and of the motivational climate in youth football: a longitudinal perspective. Percept. Mot. Skills 131, 551–567. doi: 10.1177/00315125231222152, 38096458 PMC10924548

[ref36] Sato M. Yoshida M. Wakayoshi K. Shonk D. J. (2017). Event satisfaction, leisure involvement and life satisfaction at a walking event: the mediating role of life domain satisfaction. Leis. Stud. 36, 605–617. doi: 10.1080/02614367.2016.1240221

[ref37] Scanlan T. K. Carpenter P. J. Simons J. P. Schmidt G. W. Keeler B. (1993). An introduction to the Sport Commitment Model. J. Sport Exerc. Psychol. 15, 1–15. doi: 10.1123/jsep.15.1.1

[ref38] Strauss M. E. Smith G. T. (2009). Construct validity: Advances in theory and methodology. Annu. Rev. Clin. Psychol. 5, 1–25. doi: 10.1146/annurev.clinpsy.032408.153639, 19086835 PMC2739261

[ref39] Sullivan J. Gow M. Chadwick S. (2019). China’s football dream: sport, citizenship, symbolic power, and civic spaces. J. Sport Soc. Issues 43, 493–514. doi: 10.1177/0193723519867588

[ref40] Tenenbaum G. Strauss B. Büsch D. (2007). “Applications of generalized rasch models in the sport, exercise, and the motor domains,” in Multivariate and mixture distribution rasch models, ed. Von Davier M. (New York: Springer), 347–356.

[ref41] Worthington R. L. Whittaker T. A. (2006). Scale development research: a content analysis and recommendations for best practices. Counsel. Psychol. 34, 806–838. doi: 10.1177/0011000006288127

[ref42] Wright B. D. Masters G. N. (1982). Rating Scale Analysis: Rasch Measurement. Chicago, United States: Mesa Press.

[ref43] Zeng X. He W. Guo Z. (2026a). A psychological network analysis of parental and peer support on children’s football participation: the bridging role of self-efficacy. Front. Psychol. 17:1818293. doi: 10.3389/fpsyg.2026.1818293, 42389107 PMC13319038

[ref44] Zeng X. Hu M. He W. Xie Z. (2026b). Psychosocial correlates of children’s football participation: a psychological network analysis of parental support, stereotypes, and dualistic passions. Front. Psychol. 17:1897362. doi: 10.3389/fpsyg.2026.1897362, 42453442 PMC13368343

[ref45] Zhang L. Liang Z. (2002). Athletes’ life satisfaction: the contribution of personal self-esteem and collective self-esteem. Acta Psychol. Sin. 2, 160–167.

[ref46] Ziegler M. Kemper C. J. Kruyen P. (2014). Short scales–five misunderstandings and ways to overcome them. J. Individ. Differ. 35, 185–189. doi: 10.1027/1614-0001/a000148

