# Supplementary material for: Development and psychometric validation of the Amateur Football Tournament Satisfaction Scale using Rasch analysis
Source: Front Psychol. 2026 Oct 14;17:1952185. doi: 10.3389/fpsyg.2026.1952185 (PMC13713279; doi:10.3389/fpsyg.2026.1952185)
Supplement: Supplementary file 1 [file Supplementary_file_1.docx]

# EFA AFCSS

TITLE: EFA AFCS;

DATA:

FILE = XXX;

VARIABLE:

NAMES = X1-X41;

USEVARIABLES = X7-X36;

MISSING = ALL (999);

ANALYSIS:

ESTIMATOR = MLR;

TYPE = EFA 1 6;

ROTATION = GEOMIN;

PARALLEL = 1000;

CONVERGENCE = 0.0001;

ITERATIONS = 10000;

MODEL:

OUTPUT:

STD;

TECH1;

TECH4;

# CFA AFCSS-SF

TITLE: CFA AFCSS-SF;

DATA:FILE IS XXX;

VARIABLE:

NAMES ARE X1-X61;!数据文件所有变量

MISSING=ALL(999);!定义缺失值为999

USEVARIABLE X7-X16;!本次分析用到的代码

CATEGORICAL = X7-X16;

ANALYSIS:

TYPE=GENERAL;!分析类型

ESTIMATOR=WLSMV;!内置分析方法选择最稳健的最大似然法

MODEL:

AFCSS BY X7-X16;!这个变量由哪几道题预测

OUTPUT:

STDYX MODINDICES CINTERVAL;!依次是要求结果有标准化的结果，修正指数和置信区间
