# Supplementary material for: Development and psychometric validation of the Amateur Football Tournament Satisfaction Scale using Rasch analysis
Source: Front Psychol. 2026 Oct 14;17:1952185. doi: 10.3389/fpsyg.2026.1952185 (PMC13713279; doi:10.3389/fpsyg.2026.1952185)
Supplement: Supplementary file 2 [file Table_1.docx]

**Table S1.** *English and Chinese Versions of Items: Initial Full Item Pool of the Amateur Football Competition Satisfaction Scale (AFCSS)*

| **Item** | **题目** |
| --- | --- |
| 1. The quality of the pitch turf provides pleasant visual and tactile sensations for me. | 1. 赛场的草坪质量让我感到视觉和触觉上的舒适。 |
| 6. The stadium’s audio system, announcements and music boost my competitive motivation. | 6. 赛场的音响、广播或音乐激发了我的比赛欲望。 |
| 11. Visual decorations including team flags and billboards create an immersive competition atmosphere. | 11. 赛场的布置、队旗、广告牌等视觉元素营造了浓厚的比赛氛围。 |
| 16. The enthusiasm and cheers from spectators raise my drive to compete. | 16. 观众的热情和助威声让我更有比赛动力。 |
| 21. Environmental factors such as lighting, temperature and ventilation at the venue are comfortable. | 21. 赛场的光线、温度、通风等环境条件让我感到舒适。 |
| 26. The overall competition atmosphere feels well-organized and ceremonious. | 26. 赛事整体的氛围让我感到这是一场精心组织、有仪式感的正式比赛。 |
| 2. I feel joyful taking part in this competition. | 2. 参加这场比赛让我感到快乐。 |
| 7. Emotions such as excitement and nervousness during matches make me feel fulfilled. | 7. 比赛过程中的情绪体验（如：紧张、兴奋）让我感到充实。 |
| 12. I feel proud to compete regardless of the final match result. | 12. 无论比赛结果如何，我仍为能参与这场比赛感到自豪。 |
| 17. Playing in this competition allows me to temporarily escape daily stress. | 17. 这场比赛让我暂时忘记了生活中的压力。 |
| 22. I experience emotional relief after finishing the match. | 22. 比赛结束后，我感到情绪上得到了释放。 |
| 27. I am satisfied with my personal performance in this competition. | 27. 这次赛事让我对自己的表现感到满意。 |
| 3. I have good tacit coordination with my teammates. | 3. 我与队友之间的配合让我感到默契。 |
| 8. I feel valued by my coach’s on-match guidance. | 8. 教练在比赛中的指导让我感到被重视。 |
| 13. My communications with opponents are friendly and respectful. | 13. 我与对手之间的交流是友好且尊重的。 |
| 18. The referees officiate matches in a fair and impartial manner. | 18. 裁判的执法是公平公正的。 |
| 23. I have a strong sense of belonging to my team and this competition. | 23. 我感受到自己属于这支球队和这次赛事的一部分。 |
| 28. Post-match interactions (handshakes, conversations) with teammates, opponents, referees and fans bring me pleasure. | 28. 赛后与队友、对手、裁判员、球迷的互动（如握手、交流）让我感到愉快。 |
| 4. Pre-competition notifications about time, venue and competition format are clear and timely. | 4. 赛前的信息通知（如时间、地点、赛制）是清晰及时的。 |
| 9. Supporting facilities (changing rooms, drinking stations, medical booths) are fully equipped. | 9. 赛事的场地设施（如更衣室、饮水点、医疗点）是完善的。 |
| 14. The competition’s safety measures make me feel secure. | 14. 赛事的安全保障措施让我感到放心。 |
| 19. Competition staff provide professional service. | 19. 赛事工作人员的态度是专业的。 |
| 24. Award ceremonies and group photos after matches make me feel recognized. | 24. 赛后的颁奖、合影等环节让我感到被认可。 |
| 29. The competition schedule is reasonably arranged without excessive fatigue. | 29. 赛程安排合理，不会让我感到过度疲劳。 |
| 5. I am free to play to my full potential during competitions. | 5. 在比赛中，我能按照自己的意愿自由发挥。 |
| 10. I notice improvements in my athletic performance through this competition. | 10. 我觉得自己在比赛中有所进步。 |
| 15. This competition motivates me to keep training and competing in future competition. | 15. 这次赛事让我更有动力继续训练和参赛。 |
| 20. I perceive that I hold an important role within the team. | 20. 我能感受到自己在球队中扮演着重要角色。 |
| 25. This competition helps me gain a clear understanding of my athletic ability. | 25. 这次赛事让我更清楚自己的竞技水平。 |
| 30. This competition contributes to my long-term athletic development. | 30. 我认为这次赛事对我的成长是有帮助的。 |
| *Note.* Responses were rated on a 5-point Likert scale ranging from 1 (Strongly Disagree) to 5 (Strongly Agree). | |
